# Tracking SARS-CoV-2 seropositivity in rural communities using blood-fed mosquitoes: a proof-of-concept study

**DOI:** 10.3389/fepid.2023.1243691

**Published:** 2023-12-13

**Authors:** Benjamin J. Krajacich, Djibril Samaké, Adama Dao, Moussa Diallo, Zana Lamissa Sanogo, Alpha Seydou Yaro, Amatigue Zeguime, Josué Poudiougo, Kadiatou Cissé, Mamadou Traoré, Alassane dit Assitoun, Roy Faiman, Irfan Zaidi, John Woodford, Patrick E. Duffy, Tovi Lehmann

**Affiliations:** ^1^ Laboratory of Malaria and Vector Research, NIAID, NIH, Rockville, MD, United States; ^2^ Malaria Research and Training Center (MRTC)/Faculty of Medicine, Pharmacy and Odonto-Stomatology, University of Sciences, Techniques and Technologies, Bamako, Mali; ^3^ Laboratory of Malaria Immunology and Vaccinology, National Institute of Allergy and Infectious Diseases, National Institutes of Health, Bethesda, MD, United States

**Keywords:** Africa, COVID-19, population-based epidemiology, mosquito bloodmeal, sero-surveillance

## Abstract

**Background:**

The spread of SARS-CoV-2 cannot be well monitored and understood in areas without capacity for effective disease surveillance. Countries with a young population will have disproportionately large numbers of asymptomatic or pauci-symptomatic infections, further hindering detection of infection. Sero-surveillance on a country-wide scale by trained medical professionals may be limited in a resource-limited setting such as Mali. Novel ways of broadly sampling the human population in a non-invasive method would allow for large-scale surveillance at a reduced cost.

**Approach:**

Here we evaluate the collection of naturally blood-fed mosquitoes to test for human anti-SARS-CoV-2 antibodies in the laboratory and at five field locations in Mali.

**Results:**

Immunoglobulin-G antibodies to multiple SARS-CoV-2 antigens were readily detected in mosquito bloodmeals by bead-based immunoassay through at least 10 h after feeding [mean sensitivity of 0.92 (95% CI 0.78–1) and mean specificity of 0.98 (95% CI 0.88–1)], indicating that most blood-fed mosquitoes collected indoors during early morning hours (and likely to have fed the previous night) are viable samples for analysis. We found that reactivity to four SARS-CoV-2 antigens rose during the pandemic from pre-pandemic levels. The crude seropositivity of blood sampled via mosquitoes was 6.3% in October and November 2020 across all sites, and increased to 25.1% overall by February 2021, with the most urban site reaching 46.7%, consistent with independent venous blood-based sero-surveillance estimates.

**Conclusions:**

We have demonstrated that using mosquito bloodmeals, country-wide sero-surveillance of human diseases (both vector-borne and non-vector-borne) is possible in areas where human-biting mosquitoes are common, offering an informative, cost-effective, and non-invasive sampling option.

## Introduction

**Figure 1:**
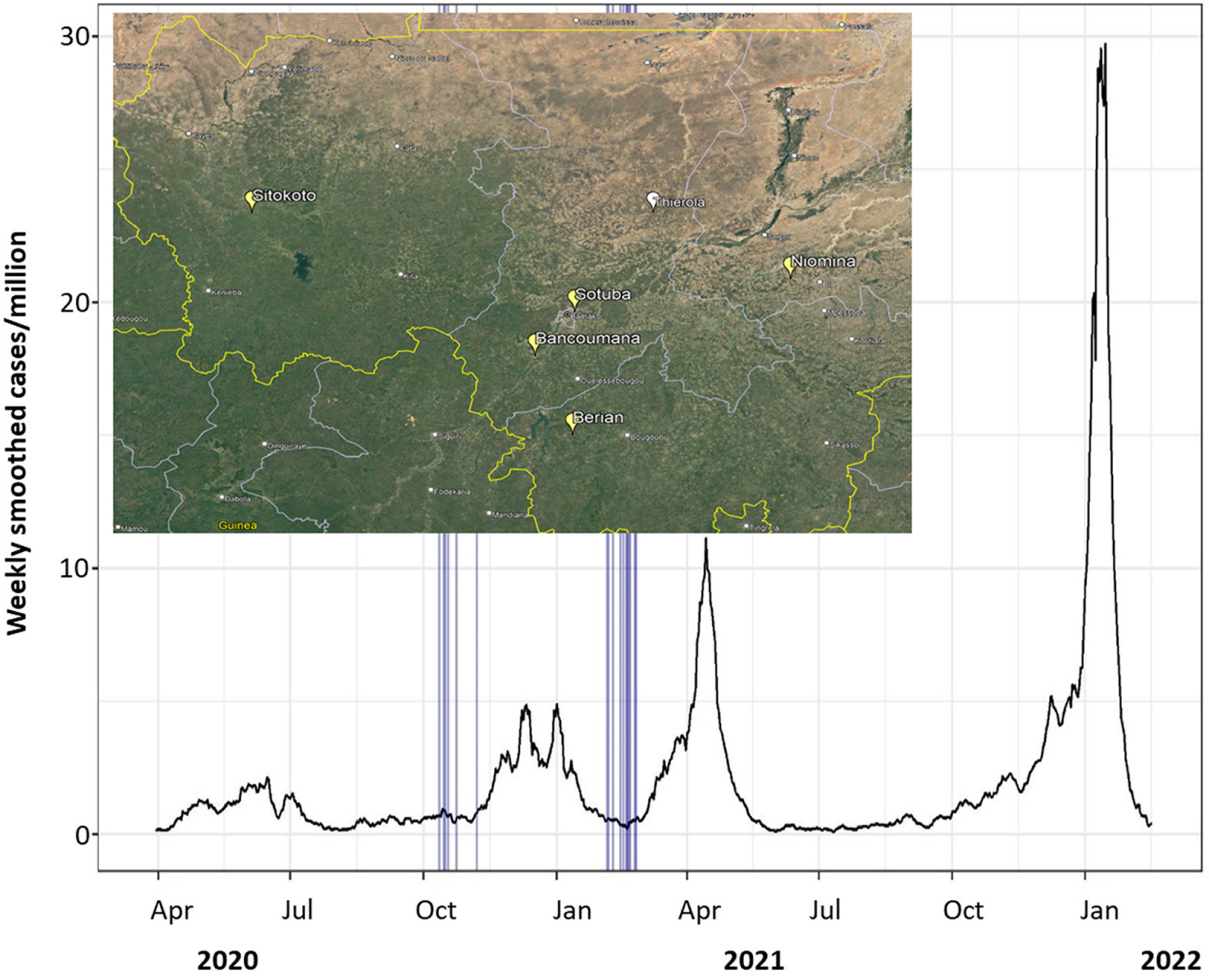
SARS-CoV-2 7-day smoothed case numbers per million people in Mali. Mosquito sampling dates across all villages marked by vertical blue lines. Data from Our World In Data (1, 2). As of 17 February 2022, 30,303 cases total in population of 20.8 million. Inset: a map showing position of communities that were sampled prior to the pandemic (Thierola, white) and during the pandemic (Yellow; map source: Google Earth).

The speed, scope, and impact of the SARS-CoV-2 virus on all corners of the globe has been unprecedented in the last 100 years, with over 662 million cases and 6.7 million deaths through 2022 (1). As of February 2022, Mali had 30,303 RT-PCR-confirmed cases of COVID-19 across four waves of infection for a population of 20.8 million (Figure 1) (2), with most cases reported from the capital, Bamako. Due to the limited testing capacity across the country, this is almost certainly a gross underestimation of the true number of infections. Sero-surveillance, in which blood samples are broadly screened for anti-SARS-CoV-2 antibodies, provides a valuable method to understand population exposure and discover the rate of spread of an infection through communities.

Enzyme-linked immunosorbent assays (ELISAs) for the detection of SARS-CoV-2 antibodies have been rapidly developed in response to the pandemic (3–5); however, population-specific assay qualification is crucial to ensure adequate test performance (6–8). We have previously reported frequent background reactivity in pre-pandemic Malian samples, and improved assay performance substantially by combining multiple antigen targets with conservative cutoffs (8). While the nature of background reactivity has not been fully explained, environmental exposures have been proposed as responsible for poor test performance in African populations compared to North American and European serum panels (6–8) including prior exposure to “common-cold” coronaviruses, such as OC43 (6), and unrelated pathogens, such as *Plasmodium* (9).

We have previously reported a sharp increase in SARS-CoV-2 seroprevalence from 10.9% in July to October 2020 to 54.7% in December 2020 to January 2021 in three communities in the greater Bamako region, with the highest rates observed in more urban areas (10). Although the sampling in this study included a town 50 km from Bamako, remote communities were not included. The expansion of sero-surveillance into more remote and rural areas of Mali may be difficult due to the requirements of trained health professionals for blood collection and analysis.

Reflecting on our experience in medical entomology, we considered mosquitoes as potential blood sampling “instruments.” In many areas of Mali, the mosquito density indoors is high, especially among species members of the *Anopheles gambiae* and *Culex pipiens* complexes, which have a strong preference for blood-feeding from humans, and a tendency to rest indoors after imbibing a bloodmeal of roughly 1–5 µl (11, 12). Previous work in this realm has found mosquito bloodmeals to have sufficient volume to detect blood-borne human pathogens (13), and various antibodies of human disease including *Trypanosoma*, *Plasmodium*, dengue virus, and Japanese Encephalitis virus antibodies (14–16). However, leveraging these insects has never, to our knowledge, been performed as a broad epidemiological sero-surveillance tool.

In this study, we evaluate the potential for anthropophilic biting mosquitoes as non-invasive blood sampling tools to measure population seroprevalence patterns, using naturally acquired SARS-CoV-2 antibodies as a proof of concept. We describe a SARS-CoV-2 serological assay suitable for use with mosquito bloodmeals, develop population-specific cutoffs to maximize specificity, characterize the durability of antibodies in the digestive environment of the mosquito midgut, and evaluate the methodology in natural settings using mosquitoes caught in five different communities in Mali, West Africa.

## Methods

### SARS-CoV-2 multiplex bead-based immunoassay and cutoff generation using pre-pandemic mosquitoes as negative controls

Due to the relatively small amount of blood drawn via a mosquito bite, a multiplex, bead-based immunological assay was selected to detect antibodies to four SARS-CoV-2 antigens simultaneously. To optimize assay performance, population-specific assay cutoffs were generated using blood-fed mosquitoes collected from the Sahelian villages Thierola and M'Piabougou, Mali, in 2017 and 2018, which had been stored on silica gel desiccant. The abdomens of individual mosquitoes were separated from the thorax under magnification using fine-tipped forceps. These abdomens were ground individually in 120 μl of sample buffer of the bead-based kit (Bio-Plex Pro Human IgG SARS-CoV-2; Bio-Rad, Irvine, CA, USA) using five to six 2.0 mm zirconia beads in a Mini-BeadBeater-96 (BioSpec Products, Inc., Bartlesville, OK, USA) at a max speed for 25 s. This slurry was spun at 13,000 *g* for 10 min to clear solids, and 50 μl of the supernatant was used in the assay according to the manufacturer’s instructions. This assay uses anti-human IgG as a detection antibody and magnetic capture beads coupled with SARS-CoV-2 nucleocapsid, receptor-binding domain, spike subunit 1 (hereafter spike1), and spike subunit 2 (hereafter spike2) viral proteins. The median fluorescence intensity (MFI) was measured per the manufacturer’s instructions. A cutoff for each antigen was calculated based on the mean MFI plus 5 SDs from a sample of 90 pre-pandemic mosquitoes (17, 18). To further enhance assay specificity, a positive test was defined as two or more antigens exceeding the cutoffs.

### Assessment of SARS-CoV-2 antibody detection in laboratory mosquitoes after direct skin feeding on volunteers

We performed direct skin feeding assays using laboratory-reared mosquitos on healthy volunteers to assess SARS-CoV-2 antibody detection in a controlled environment, define the window of time antibodies can be reliably detected in mosquito bloodmeals, and determine if dried mosquitoes are suitable for the assay. All aspects of the work involving human volunteers were approved by the Ethics Committee in the University of Bamako as part of the institutional review board protocol (No. 2020/78/CE/FMOS/FAPH).

*Anopheles coluzzii* mosquitoes were reared as previously described (19). Briefly, colonized, disease-free mosquitoes were reared in plastic trays (30 cm × 25 cm × 7 cm) with 1.5 L of dechlorinated water. Larvae were fed with yeast supplement in the first 24 h after larval emergence and fish food until emergence as adults. These adult mosquitoes were held until they were 3–5 days old, at which point they were starved overnight and allowed to feed upon human volunteers and stored as described below. Two volunteers who had recovered from a PCR-diagnosed SARS-CoV-2 infection and one volunteer with no known SARS-CoV-2 infection were fed upon by groups of 100 laboratory-reared, disease-free *An. coluzzii* mosquitoes. Mosquitoes were kept under normal insectary conditions for set timepoints after feeding (0, 2, 4, 8, and 30 h) to analyze the effect of digestion on recoverability of the antibodies. At each timepoint, a subset of 10 mosquitoes were killed and stored on a small piece of cotton ball in a 1.7 ml Eppendorf tube with silica gel desiccant (#13767; Millipore Sigma, Burlington, MA, USA). Mosquitoes were kept on the desiccant for 1 week at room temperature before storage at −80°C until the analysis as described above.

To validate the results of the pilot experiment and further evaluate the ability to distinguish between COVID-exposed (seropositive) and COVID-naïve (seronegative) individuals using the mosquitoes that fed on them, a cohort of 13 volunteers was used. This cohort consisted of residents (all aged >16 years) of the rural village of Thierola with an unknown SARS-CoV-2 status. Of this cohort, two (VCT and VKT; “V” stands for volunteer, “C” and “K” as a unique identification, and “T” for the community) had worked in a mine during several months before the experiment and two lived in cities (“H” in Bamako and “M” in Kita). An additional volunteer, a resident of Bamako, was vaccinated against SARS-CoV-2 3 months before the experiment. Each volunteer was fed upon by groups of 50 laboratory-reared, disease-free *An. coluzzii* mosquitoes. In this cohort, mosquitoes were held for 0, 5, 10, or 30 h after feeding and stored and tested as described. For volunteers with an unknown SARS-CoV-2 status, we assessed putative seropositivity based on the number of blood-fed mosquitos with SARS-CoV-2 antigen reactivity above the threshold and the number of antigens above the threshold.

To evaluate assay performance and performance over time after mosquito bloodmeal in our pilot cohort, we created 100 sample splits for each timepoint using bootstrapping using the “rsample” R package (20). From these splits, we estimated test sensitivity and specificity from the putative positivity of the volunteers with the “yardstick” R package (21). The overall test sensitivity and specificity was estimated based on 5 and 10 h timepoints as these are the most likely range of time periods after feeding to capture mosquitoes (i.e., morning collection after a likely nocturnal feeding window).

### Wild-caught mosquito-based sampling in Malian communities

Similar to the pre-pandemic mosquito collection in Sahelian villages (above), indoor resting mosquitoes were collected by aspiration from 40 houses in each of five communities (latitude and longitude are in parentheses): Bancoumana (12.20862, −8.2646); Berian (11.4197, −7.9351); Nionina (12.9873, −5.997231); Sitokoto (13.637307, −10.818615); and Sotuba (12.66181, −7.91915), Mali (Figure 1). These houses were chosen to have at least one occupied bedroom, to have given permission for sampling via the homeowners, and to be spread across the community with a minimum of 50 m between them (20 m in the smaller villages). The sampling of blood-fed mosquitoes was performed during October and November 2020 and February 2021 with handheld aspirators in the morning (07:00–10:00). Mosquitoes were desiccated on silica gel and stored at −20°C until shipment where they were stored at −80°C until analysis.

### Bloodmeal host determination: Human vs. animal blood

Mosquito host feeding sources were determined through a qPCR high resolution melt-curve analysis targeting cytochrome B gene fragment, following published protocols (22). Briefly, DNA was extracted from 5 μl of the above mosquito slurry by combining it with 20 μl of QuickExtract solution (Lucigen), and incubated for 65°C at 15 min with a final inactivation of 98°C for 2 min. From this, 2 μl of extract was combined with 5 μl SsoAdvanced Universal SYBR Green Supermix, 2 μl water, and 1 μl of 10 μM CytB primers, and analyzed with the published amplification/melt conditions in a Mic qPCR Cycler (Bio Molecular Systems, Australia). Bloodmeal discrimination for the purposes of this paper was classified as human if the melting temperature of the amplified product fell between 85.75°C ± 2°C, and otherwise considered non-human if the melt rate (−dF/dT) peaks fell outside this range.

### Estimation of community SARS-CoV-2 seroprevalence over time

Samples of approximately 60 blood-fed mosquitoes per village per timepoint as well as 90 blood-fed mosquitoes collected before the pandemic (pre-pandemic, above) were processed and analyzed for SARS-CoV-2 antibodies as described. Pre-pandemic samples collected from similar rural communities in the Sahel of Mali (Figure 1) used to generate assay cutoffs were included to estimate baseline community seroprevalence. Where possible, mosquitoes were sampled from the same houses across time periods to assess the progression of seropositivity in a semi-matched population. The population was considered semi-matched due to uncertainty whether the individual the mosquito fed on is a member of that house, and if so which individual it was. It was assumed that each blood-fed mosquito fed randomly, and collection of multiple mosquitoes in the same house may allow to compare data at the individual mosquito and house levels. To test the difference between quantiles of pre-pandemic and pandemic distributions of reactivity to different antigens, we used quantile regression implemented by Proc Quantreg (23), which extends the general linear model for estimating conditional change in the response variable across its distribution as expressed by quantiles, rather than its mean (though the median is similar to the mean in symmetric distributions). Quantile regression does not assume parametric distribution (e.g., normal) of the random error part of the model, thus it is considered semi-parametric. The benefit of this analysis is that it addresses changes in reactivity to antigens that could be detected in the higher quantiles even when the mean or the median are less affected, without imposing cutoffs. In fact, it can be used to estimate if there is a monotonic increase over time (and over quantiles) and estimate the seroprevalence change (from the pre-pandemic baseline) per antigen. The parameter estimates in linear quantile regression models are interpreted as in typical general linear model (GLM), as rates of change adjusted for the effects of the other variables in the model for a specified quantile (24).

### Seropositivity adjustments

The proportion of seropositive bloodmeals (hereafter also seropositive mosquito) was estimated and stratified by time and site with 95% CIs calculated using the “yardstick,” “infer,” and “rsample” packages (20, 21, 25) with 1,000 bootstrap replicates. To improve the accuracy of community seroprevalence estimates, crude seropositivity results were adjusted in three ways. First, the assay performance was adjusted using the following formula incorporating sensitivity and specificity estimates derived from the 5 and 10 h timepoints after the controlled mosquito skin feeding cohort experiment (above) (26).

$$\mathrm{Adjusted}\;\mathrm{Seroprevalence}=\;\frac{\mathrm{crude}\;\mathrm{seroprevalence}+\mathrm{specificity}-1}{\mathrm{sensitivity}+\mathrm{specificity}-1}.$$

Second, we adjusted the seroprevalence estimates to account for non-human bloodmeals at each collection by adjusting the total number of samples tested by the estimated proportion of non-human bloodmeals. Finally, seroprevalence when sampling a single mosquito per house was estimated across 1,000 random draws of one mosquito per house per time period. For adjusted seroprevalence estimates, 95% CIs were generated as described with 1,000 bootstrap replicates.

## Results

### SARS-CoV-2 bead-based immunoassay cutoff generation using pre-pandemic mosquitoes as negative controls

**Figure 2:**
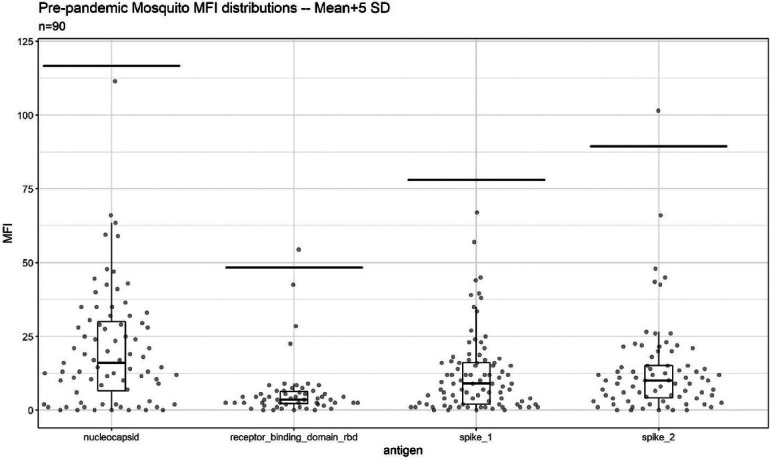
Background reactivity of 90 blood-fed mosquitoes collected via indoor aspiration prior to SARS-CoV-2 emergence and stored desiccated on silica gel until analysis. Per antigen cutoffs are marked via line. The two points above cutoff were single antigen positive mosquitoes.

Assay cutoffs were generated based on pre-pandemic, silica gel stored, blood-fed mosquito samples (*n = *90). Median fluorescent intensity (MFI) values per antigen were non-normally distributed (Shapiro–Wilk normality test, *p *< 0.001 for all antigens). The cutoffs generated using negative control mosquitos were as follows: N: 116.7; RBD: 48.3; S1: 78.0; and S2: 89.4, lower than the manufacturer suggested MFI cutoffs for 1:100 dilution of serum (N: 450, RBD: 250, S1: 250, and S2: 750). In pre-pandemic mosquitoes, single antigen positivity was uncommon (*n *= 2, 2.2%) (Figure 2 and Supplementary Table S1). No pre-pandemic mosquitoes were seropositive for two or more antigens.

### SARS-CoV-2 antibody detection over time in laboratory mosquitoes fed on human volunteers

In an initial proof-of-concept time-course experiment, mosquitoes were held for set timepoints after blood-feeding on one negative control and two positive control volunteers to assess the detectability of the antibodies in the bloodmeal during mosquito digestion under normal insectary conditions. Consistent with previous studies on antibodies against other pathogens (13–16, 27, 28), antibodies against SARS-CoV-2 remained detectable at least 10 h after feeds, with a loss of signal seen between that timepoint and 24 h later (data not shown).

**Figure 3:**
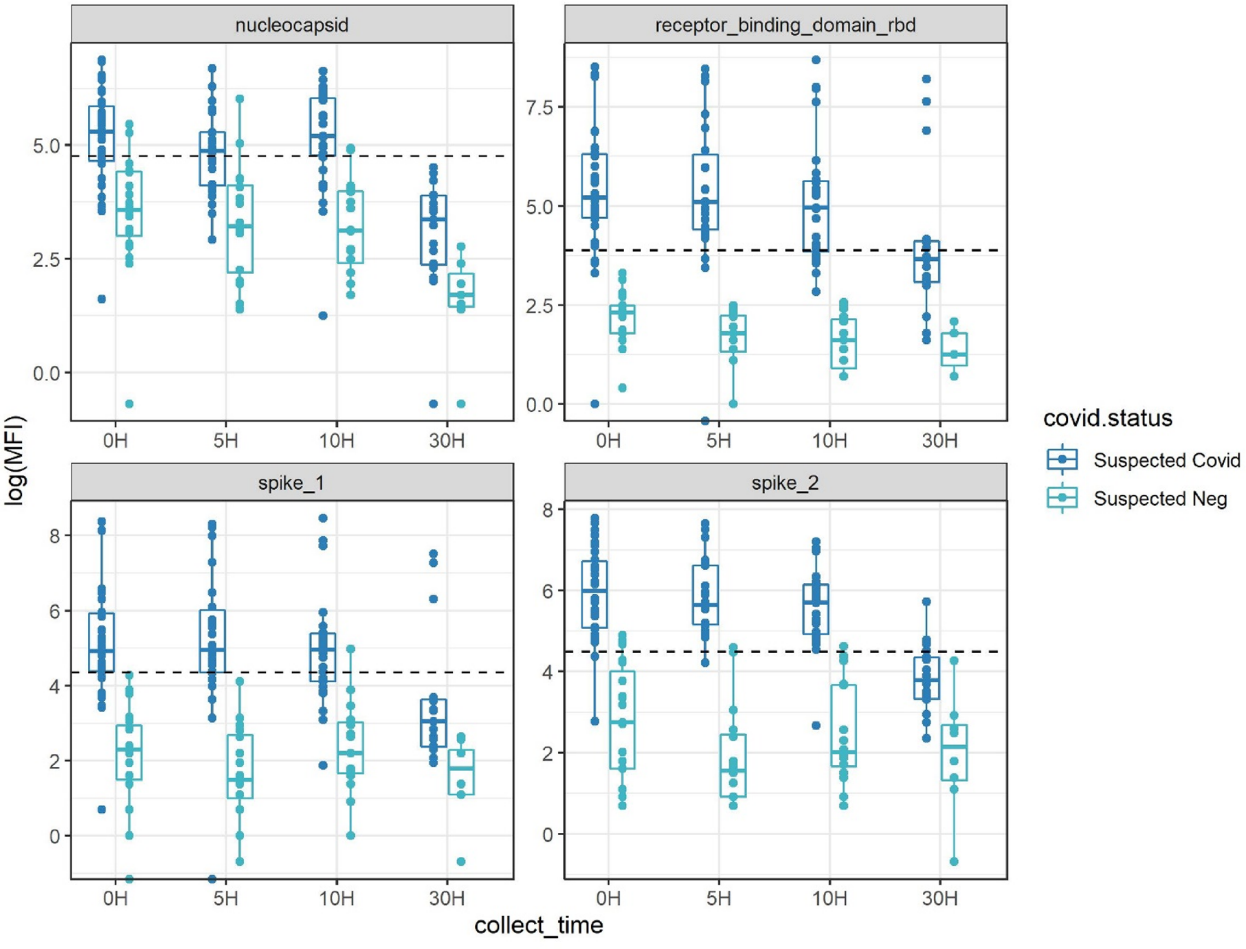
Antibody detectability above antigen-specific cutoffs (dotted line) with the four-antigen multiplex immunoassay after set periods of digestion post-blood-feeding on human volunteers. Suspected COVID-19 had 2+ antigens over pre-pandemic cutoffs at multiple timepoints, suspected negative had a maximum of one antigen positive at any timepoint. Inconclusive (based on a single mosquito bloodmeal) had one mosquito with two antigens positives at various timepoints (see also Supplementary Figures S1, S2).

In a larger cohort of 13 volunteers with unconfirmed SARS-CoV-2 infection history undergoing direct skin feeding, higher assay signals were observed in mosquitos processed within 10 h of feeds, with many exceeding assay cutoffs (Figure 3, Supplementary Figures S1, S2). Based on the positivity rate of fed mosquitoes at timepoints 0, 5, and 10 h (Figure 3 and Supplementary Figure S1), eight of 13 volunteers were considered likely to be SARS-CoV-2 seropositive. VAT received the COVID-19 vaccination 3 months before the experiment. All mosquitos fed on VAT demonstrated pronounced reactivity to three or more SARS-CoV-2 antigens exceeding cutoffs up to 30 h after blood-feeding (Supplementary Figure S3). At the 0, 5, and 10 h post-feed timepoints, all mosquitos that fed on VBT and VDT were positive (reactivity exceeding threshold for two or more antigens in 11/11 and 12/12 mosquitoes, respectively). Similarly, high rates of mosquito positivity were observed across these post-feed timepoints for VFT, VIT, VLT, VMT, and VGT (10/11, 9/10, 4/5, 9/10, and 8/10, respectively). As a result, these eight individuals were considered true positives, with negative mosquitoes considered false negatives. In contrast, volunteers VHT, VCT, VET, and VJT were considered seronegative (0/11, 0/12, 0/7, and 0/10, respectively). Notably, in VKT one of 12 mosquitos was positive (Figure 3 and Supplementary Figure S1), indicating either a false-positive mosquito or result near the limit of detection. Using these putatively positive and negative volunteers as reference sources for mosquito feeds, assay misclassification varied between antigens (in timepoints 0, 5, and 10 h), being highest in nucleocapsid with 12% false positive and 34% false negative (*N* = 132 mosquitoes), followed by spike1 (2% false positive and 30% false negative, *N* = 132), RBD (0% false positive and 17% false negative, *N* = 132), and spike2 (10% false positive and 6% false negative, *N* = 132). Thus, using reactivity against multiple antigens for a diagnosis of a suspected seropositive is needed to overcome these rates of misclassification based on a single antigen.

Based on these classifications of volunteer infection status and the requirement that reactivity above cutoff must be observed for two or more antigens, we estimated the sensitivity and specificity of our assay over time (Table 1).

**Table 1:** Sensitivity and specificity estimations during each timepoint post-feeding a total of 159 mosquitoes on 13 volunteers (Figure 3 and Supplementary Figure S1).

Time post-feed (h)	Mean sensitivity	95% CI sensitivity	Mean specificity	95% CI specificity
0	0.932	0.807–1	1	1–1
5	0.871	0.719–1	1	1–1
10	0.954	0.882–1	0.942	0.833–1
30	0.288	0.103–0.474	1	1–1
5 and 10	0.925	0.826–0.980	0.975	0.903–1

95% CI were calculated using bootstrapping. A 5 and 10 h merged timepoint estimate (5 and 10 h) was calculated as these are the more likely timepoints for collection of blood-fed wild mosquitoes post-feeding (than 0 or 30 h).

While sensitivity was high at the 0, 5, and 10 h timepoints (0.871–0.954), it dropped substantially at 30 h, with only mosquitoes from two of the previously infected volunteers with the highest overall MFI values showing positivity at this timepoint (Supplementary Figure S2). The sensitivity and specificity for the combined 5 and 10 h timepoints, considered most representative of field-caught mosquitos, were 92.5% and 97.5%, respectively.

### Sero-surveillance of Malian communities

A total of 579 blood-fed mosquitoes (252 were *An. gambiae* s.l. and 327 were *Culex* spp.) collected indoors in five Malian communities were analyzed. Analyzed mosquitoes were subsampled to be largely consistent between sampling periods (284 in October–November 2020, 295 in February 2021), villages (118 in Bancoumana, 108 in Berian, 115 in Nionina, 118 in Sitokoto, and 120 in Sotuba), and total houses sampled per village (44 in Bancoumana, 35 in Berian, 35 in Nionina, 34 in Sitokoto, and 33 in Sotuba).

**Figure 4:**
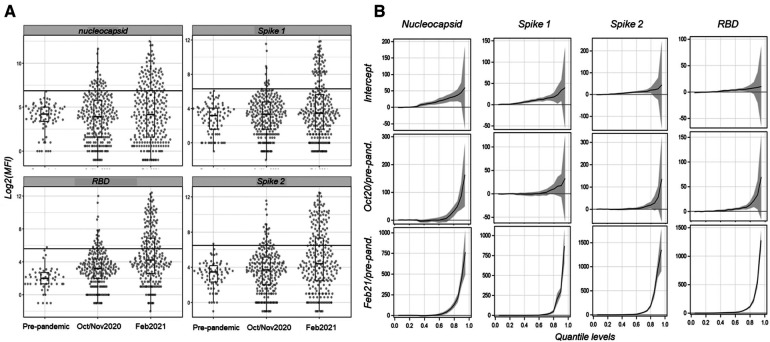
Distributions of the MFI per antigen comparing pre-pandemic (*N* = 90) and pandemic mosquitoes (October–November 2020, *N* = 284; and February 2021, *N* = 295) and quantile regression results showing quantile-specific changes in reactivity over time for each antigen. (**A**) Reactivity distribution of each antigen and time period overlaid with box-whisker plots. The cutoffs are shown by the horizontal lines. (**B**) Results of quantile regression models fitted to each antigen with period as the independent variable, showing the intercept and the effect of each pandemic time period relative to the pre-pandemic baseline with 95% confidence interval (gray band). Line segments above zero indicate quantiles in which the effect is positive and statistical significance is indicated if the CI range does not overlap with the zero baseline.

To ensure that changes in seroprevalence are independent of our cutoff values and definition of positivity, we first evaluated changes in reactivity over time. An increase in reactivity from the pre-pandemic baseline was apparent across all four SARS-CoV-2 antigens (Figure 4). Because we suspected that only a fraction of the population would be seropositive, we used quantile regression to evaluate which quantiles have changed and if the change was consistent across quantiles. An advantage of this approach is that it does not assume cutoffs (see “Methods”), yet it can be used to estimate the quantiles of the population that exhibits crude reactivity changes over the pandemic for each antigen. Considering nucleocapsid and spike-1, an increase over the pre-pandemic baseline was significant at October–November 2020, starting from the 80th and 75th quantiles, respectively (quantile regression, *t_df_*_ = 1 _= 2.74 and 2.14, *p* = 0.006 and *p* = 0.033, respectively) and increasing in significance at higher quantiles, whereas at February–March 2021, a significant increase was detected from the 65th and 70th quantiles, respectively (quantile regression, *t_df_*_ = 1 _= 2.71 and 2.49, *p* = 0.007 and *p* = 0.013, respectively) (Figure 4B). In RBD, an increase over the pre-pandemic baseline was significant at October–November 2020, starting from the 50th quantile (*t_df_*_ = 1 _= 3.17, *p* = 0.002) and increasing in significance at higher quantiles, whereas at February–March 2021, a significant increase was detected from the 30th quantile (*t_df_*_ = 1 _= 2.89, *p* = 0.035) (Figure 4B) and increased thereafter. In spike-2, an increase over the pre-pandemic baseline was significant at October–November 2020, starting from the 70th quantile (*t_df_*_ = 1 _= 2.53, *p* = 0.012) and increasing in significance at higher quantiles, whereas at February–March 2021, a significant increase was detected from the 50th quantile (*t_df_*_ = 1 _= 2.43, *p* = 0.016) (Figure 4B) and increased thereafter. These results, at the single antigen level, exhibited relative change over time, and thus, support a consistent increase from the pre-pandemic baseline. On average, across antigens and the five communities, these estimates support 31% crude seroprevalence in October–November 2020, which increased to 46% in February–March 2021. Moreover, the increase in the magnitude of the reactivity (Figure 4) indicates that a fraction of the population has experienced multiple exposure events resulting in elevated titers among sero-positives (aside from the greater fraction of the population showing an increase over the pre-pandemic levels). The crude daily rate of infection was estimated by the difference in mean prevalence (across antigens in the whole population) between timepoints divided by the median number of days between samples, which was 0.13%/day between October–November 2020 and February 2021 following Sagara et al. (10). Assuming that COVID-19 started spreading in the country 1 week before the discovery of the first case(s) in Mali (25 March 2020; http://www.xinhuanet.com/english/2020-03/25/c_138916218.htm), the crude daily infection rate between this and the October sample was 0.15%/day.

**Figure 5:**
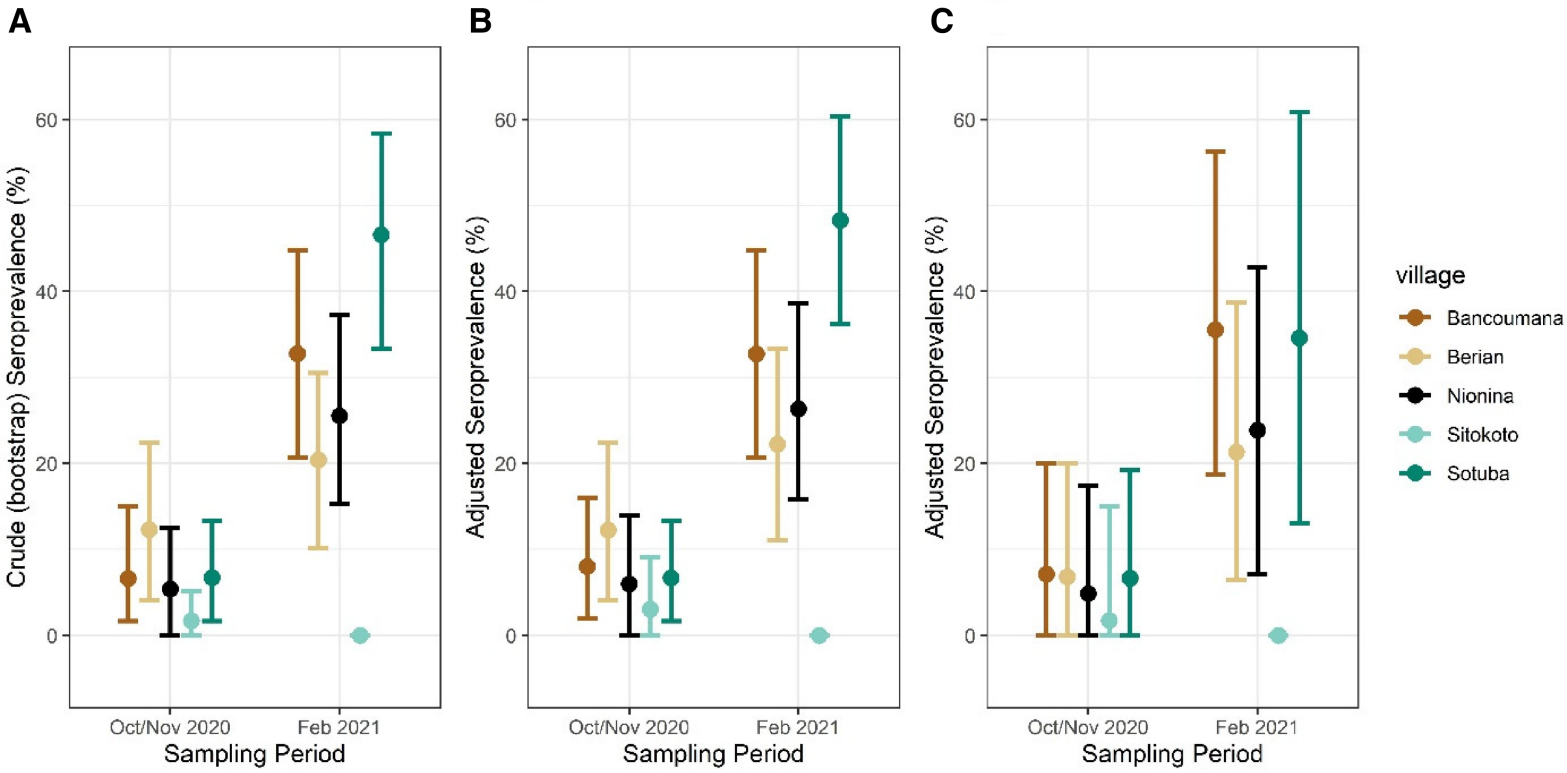
Crude (**A**) and adjusted seroprevalence per sampling village. Adjusted seroprevalence for test sensitivity and bloodmeal composition (**B**), and test sensitivity with one mosquito per sampling period per household (**C**). Mean seroprevalence per village/period with 95% CIs shown.

Crude seroprevalence increased in four of the five communities between October–November 2020 and February 2021 (Figure 5A and Supplementary Figure S3). Notably, no change was recorded for the village Sitokoto, which had a very low seroprevalence (1.8%) in October–November 2020 (Figure 5A).

The crude serological data indicate marked heterogeneity over time as well as in space (Table 2). Considering a house “positive” if it had at least one positive mosquito, seroprevalence at the house level was typically higher than that at the mosquito level, yet the differences over time and across villages were consistent as were the statistically significant differences between periods and across villages (Table 2). Significant differences between villages were detected only in the February 2021 period (Table 2). At the range of seroprevalence measured, there was a consistent relationship with house seroprevalence 50% higher than that at the mosquito level (Supplementary Figure S4, Discussion).

**Table 2:** Crude seroprevalence (*N*) over time across spatial scales and sampling units.

Spatial scale	Unit	October–November 2020	February 2021	*p* (homogeneity test over time)	*p* (homogeneity test across villages)
Overall Overall	Mosquito House	4% (284) 6.7% (119)	20% (295) 30.1 (143)	0.0001, *χ*^2^*_df_*_ = 1 _= 35.4 0.0001, *χ*^2^*_df_*_ = 1 _= 22.6	
Bancoumana Berian Nionina Sitokoto Sotuba Cross villages	Mosquito Mosquito Mosquito Mosquito Mosquito Mosquito	5% (60) 8% (49) 2% (56) 2% (59) 3% (60) *p* = 0.4, *χ*^2^*_df_*_ = 4_ = 4.1	31% (58) 12% (59) 17% (59) 0% (59) 40% (60) *p* = 0.0001, *χ*^2^*_df_*_ = 4 _= 36.9	0.0002, *χ*^2^*_df_*_ = 1 _= 13.6 0.5, *χ*^2^*_df_*_ = 1_ = 0.4 0.006, *χ*^2^*_df_*_ = 1 _= 7.6 0.3, *χ*^2^*_df_*_ = 1 _= 1 0.0001, *χ*^2^*_df_*_ = 1 _= 23.7	Breslow–Day test for homogeneity: 0.006, *χ*^2^*_df_*_ = 4 _= 14.4
Bancoumana Berian Nionina Sitokoto Sotuba Cross villages	House House House House House House	10% (30) 5% (20) 4% (23) 5% (20) 8% (26) *p* = 0.91, *χ*^2^*_df_*_ = 4 _= 0.95	50% (32) 19% (31) 29 (28) 0% (29) 57% (23) *p* = 0.0001, *χ*^2^*_df_*_ = 4 _= 27.9	0.0002, *χ*^2^*_df_*_ = 1 _= 11.7 0.15, *χ*^2^*_df_*_ = 1 _= 2.1 0.024, *χ*^2^*_df_*_ = 1 _= 5.1 0.41, Fisher exact test 0.0002, *χ*^2^*_df_*_ = 1 _= 13.7	Breslow–Day test for homogeneity: 0.023, *χ*^2^*_df_*_ = 4 _= 11.3

Although the indoor resting mosquitoes caught for this study are known to feed predominantly on humans (11, 29–32), we assessed the variation among villages and timepoints in this trait, which could confound our results because our secondary antibody was anti-human IgG (see “Methods”). A bloodmeal analysis to identify human and non-human hosts (see “Methods”) was performed on 221 mosquitoes. Overall, 88% fed on human blood, including 9% that fed on human and other animal blood (mixed, Supplementary Figure S5). The overall human feeding rate was lower in *Anopheles* (79%, *N* = 109) than in *Culex* (97%, *N* = 112, *p* = 0.001 *χ*^2^*_df_*_ = 1_ = 18.1), was similar between timepoints (86% in October–November 2020 vs. 91% in February 2021, *χ*^2^*_df_*_ = 1_ = 1.83, *p* = 0.17), and was in the range of 66% (Sitokoto) to 97% (Sotuba) among villages (*χ*^2^*_df_*_ = 4_ = 27.3, *p* = 0.0001) (Supplementary Figure S5). With the other villages’ blood-feeding rate on humans greater than or equal to 91% (*χ*^2^*_df_*_ = 4_ = 1.6, *p* = 0.6), only the mosquitoes from Sitokoto exhibited an exceptionally low human feeding rate. To accommodate the variation in the blood-feeding rate on our seroprevalence rates, we adjusted the seroprevalence data in each village and timepoint to the fraction of mosquitoes that fed on humans (Figure 5B).

The numbers of mosquitoes collected and analyzed per house across the five villages in each time period varied (medians = 2 and 3, maximum = 11 and 49, respectively) (Supplementary Figure S6), and thus we calculated the human seroprevalence per village using a bootstrap subsampling of one mosquito per house per village per time period (Figure 5C). This led to similar point estimates of prevalence, though with a wider 95% CI.

## Discussion

This is the first study evaluating the use of serological data derived from blood-fed mosquitoes to measure the spread of a non-vector-borne disease, namely, COVID-19, at a country scale. This approach has a high potential to fill the gap where capacity to effectively sample the target human (host) population directly is low, but where mosquitoes that feed on people are abundant—settings that are common in many developing countries. As this was a proof-of-concept evaluation of this approach, rather than a full-scale investigation (in preparation), we have limited the number of communities, time periods, and samples analyzed. Yet, the results reveal a sharp increase in exposure to SARS-CoV-2 between October 2020 and February 2021, albeit not across all communities. Furthermore, a comparison of key patterns detected here with those established using the classic sero-surveillance study in some of the same Malian communities (8) suggests high congruency (below) (10). Overall, our results demonstrate that this approach provides valuable insights as to the magnitude of human exposure and its variation over space and time, which can inform epidemiological assessments and decisions.

However, this approach does not convey individual patient exposure status (or seroconversion in repeated sampling) because the individual person the blood came from remains unknown as is the information about their age, sex, etc. Uncertainties regarding the exact volume of the sera a mosquito imbibes, the exact time since blood-feeding, and especially whether a mosquito fed on a human or animal host, and the fraction of mosquitoes that fed on the same people preclude interpreting “mosquito seroprevalence” as identical to the human population's seroprevalence, without accommodating additional information. Finally, the small volume of blood available in a mosquito (typically 1–5 μl (12)) limits the number of serological assays that can be performed on a single sample. Below, we consider these factors in the analysis and interpretation of the results on the spread of SARS-CoV-2 in Mali and in similar application of this approach in the future for this or other diseases.

The prerequisites for using blood-fed mosquitoes for serological studies include establishing the dynamics of antibody detection over time since blood-feeding (using the same preservation method and conditions used in the field) and reactivity cutoffs that are validated using direct feed on local volunteers or blood from seropositive and seronegative individuals from the target populations (6–8). Early experiments to evaluate the effect of time after feeding on antibody detection revealed that mosquitoes preserved in 80% ethanol indicated rapid reactivity degradation compared with those desiccated on silica gel (not shown). Both laboratory experiments in NIH and field studies in Mali confirmed earlier studies (13, 15, 16, 27, 28) that antibody detection persisted with minimal degradation until at least 10 h and degradation was evident at later timepoints (24–36 h after feeding) (see, for example, Figure 3). Since most *Anopheles spp.* and *Culex spp.* mosquitoes bite late at night (22:00–04:00) (31, 33–37) and mosquito collection took place between 07:00 and 10:00, most mosquitoes were killed and preserved 3–11 h after feeding. Moreover, we separated freshly fed mosquitoes that were subjected to serological analysis from later stages of blood digestion, including semi-gravid and gravid or non-fed mosquitoes. We established reactivity cutoffs per antigen with wide margins based on the mean and 5 SDs, using pre-pandemic, silica gel stored blood-fed mosquito samples from the target population that represent natural background reactivity (Figure 2). The low single antigen positivity (2.2%) in the pre-pandemic mosquitoes (Figure 2 and Supplementary Table S1) was further minimized by requiring that seropositive mosquitoes exhibit reactivity above cutoffs in two or more antigens. These cutoffs were tested in a trial with 13 volunteers, whose infection history with SARS-CoV-2 was unknown (except for one). Based on the highly consistent seropositivity of the mosquitoes that fed on them (mosquitoes/volunteer >10), the volunteers were readily classified into putative positive and negative states using high consensus among mosquitoes (in timepoints 0, 5, and 10 h) (Figure 3 and Supplementary Figures S1, S2). Likewise, despite moderate misclassification by single antigens (up to 12% false positive and 34% false negative among *N *= 132 mosquitoes in NC) (8, 38), considering the two-antigen definition at the 5 and 10 h post-feeding timepoint (above), only 2.5% were false-positive mosquitoes and only 7.5% were false-negative mosquitoes, assuming the classification of individuals was correct (Table 1). A larger sample size of volunteers would likely provide narrower confidence intervals for these estimates.

Overall, the results based on 669 blood-fed mosquitoes collected indoors across five Malian communities (Bancoumana, Berian, Nionina, Sitokoto, and Sotuba following collection in two pre-pandemic villages) revealed an increase in reactivity from the pre-pandemic baseline across all four SARS-CoV-2 antigens (Figure 4). This increase was significant between the pre-pandemic and the early (October–November 2020) and late (February 2021) pandemic time periods (Figures 4, 5), but also between the early and late pandemic time periods (quantile regressions, *p* < 0.01) (Table 2). Assuming minimal change in confounders such as human feeding rate, this trend presents a compelling proof for the utility of a mosquito-based analysis of disease spread, especially because it does not depend on cutoff values. This analysis indicated a steady increase of the fraction of the population exhibiting elevated reactivity over the pre-pandemic level as well as elevated intensity of the reactivity across the higher quantiles (Figure 4), suggesting higher titers among putative positives, as expected if people are repeatedly infected when more individuals carry the virus. Assuming these five, mostly rural, communities represent the whole of Mali, the crude daily rate of infection (estimated by the difference in mean prevalence across antigens between timepoints divided by the median number of days between samples as explained above) was 0.13%/day between October–November 2020 and February 2021. Assuming that COVID-19 started spreading in the country 1 week before the discovery of the first case(s) in Mali (above), the crude daily infection rate between this and the October sample was 0.15%/day. Albeit lower than those reported from Mali (8, 10), the difference may reflect the more remote and rural settings of the communities sampled here. Indeed, at Doneguebougou, the most rural community sampled by Sagara et al. (10), which is located approximately 15 km from Bamako, their estimate for the same time period was similar (0.19%/day), and unlike our estimate, their rate included individuals who were positive at the first timepoint and negative in the second timepoint. A similar approach to detect immunoglobulin M (IgM, instead or together with IgG) in mosquito bloodmeals may be used to estimate the recent exposure rate of infection. The optimization and validation of IgM bloodmeal assays would be required.

Following the definition of a seropositive mosquito's bloodmeal (reactivity > cutoff in two or more SARS-CoV-2 antigens), we estimated the crude population seroprevalence of each community and timepoint, assuming each mosquito fed on a randomly selected resident (Figure 5 and Supplementary Figure S3). The seroprevalence at the house level was 50% higher than that at the mosquito level (Table 2 and Supplementary Figure S4), reflecting the combined effects of the clustering of seropositive individuals between houses in a village, the number of mosquitoes analyzed per house, and the fraction mosquitoes that blood fed in one house overnight and moved into another by morning (39, 40). Because of this and the quicker saturation of the house seroprevalence (defined as having at least one seropositive mosquito in a house at a given time period), we suggest that the crude seropositivity at the mosquito level provides more accurate estimate of the community true seroprevalence. In addition, blood-fed mosquitoes should be sampled from at least 25 houses in the community, and possibly from a larger number based on its total size, spatial organization, and heterogeneity with respect to relevant factors, e.g., proximity to school, market, and so on. Overall, the crude seroprevalence rate in October–November 2020 was 6.5% [Sitokoto: 1.8% (minimum), Berian: 12.2% (maximum)] (Figure 5A, Table 2), representing 7 months after the discovery of the first case of COVID-19 in Mali. However, 3.5 months later (February 2021), the overall crude seroprevalence was dramatically higher at 25.0% [Sitokoto: 0% (minimum), Sotuba: 46.5% (maximum)] (Figure 5A, Table 2). This rise corresponds to the first peak of elevated transmission in Mali (November 2020–January 2021) (Figure 1).

Our crude seroprevalence may underestimate actual human population seroprevalence because the assay's sensitivity was lower than its specificity (Table 1), while the majority of the population would still be seronegative and because some of the mosquitoes had taken their bloodmeal on non-human hosts (which our ELISA cannot detect even if that host had antibodies against SARS-CoV-2). The adjusted seroprevalence values were typically 2% higher than the crude seroprevalence across communities, and in each one, except in Sotuba during February 2021, where the adjusted seroprevalence was 4.5% higher than the crude value (Figure 5). Overall, 12% of mosquitoes fed on non-human blood (*N* = 221), aside from 9% that fed on human and other animal blood, proportions that are consistent with previous studies (11, 29, 31, 34, 35). None of the mosquitoes that fed on animal blood were seropositive (*N* = 26; one mosquito was reactive to a single antigen). A minor difference was detected between October–November 2020 (86%) and February 2021 (91%, above) and feeding on human blood was above 91% in all villages except in Sitokoto (66%), which also had the lowest crude seroprevalence. Incorporating a bead that indicates human IgG or other human-specific antigen into a single ELISA would be helpful in future studies, especially in areas where feeding on non-human hosts is more common. This assay could be further improved by incorporating an antigen to capture IgG for a common pathogen, such as malaria as a positive control (as long as quantity of the sera in the bloodmeals is sufficient). The total IgG will ensure the target is available and possibly to standardize the reactivity to particular antigens, whereas the detection of the common pathogen (in accordance with expectations) will further validate the assay results. Ideally, including the antigens of multiple pathogens can further increase the value of this approach. Because most populations have been exposed to SARS-CoV-2 and/or vaccinated against it, that assay may be reserved for comparing isolated communities in remote areas or following its spread across new host species (e.g., primates if mosquitoes feeding on these hosts are available). Moreover, this approach could be powerful to address rare and emerging pathogens in remote areas after the assay is optimized to detect antibodies against these pathogens in and around enzootic foci. Using a new serological assay based on mosquito bloodmeals would benefit from a comparison with conventional serology on one or few communities. Different mosquito species or ecological conditions leading to feeding on non-human (non-target) hosts can be directly estimated and the seroprevalence results adjusted accordingly (as done here). When sampling strongly anthropophilic mosquitoes (e.g., *An. gambiae s.l.*, *Cx. quinquefasciatus*, *Ae. aegypti*), crude seropositivity may serve as a first-order approximation of the population seroprevalence, until validation can be incorporated. Samples of desiccated blood-fed mosquitoes can be used for such assays years after collection, allowing the mining of historical epidemiological events and processes. Finally, to consider the possibility that mosquitoes collected in the same house fed on the same person, we also estimated the human seroprevalence by resampling one mosquito from each household (Figure 5C). Because most houses had >3 occupants, and the number of mosquitoes analyzed from the same house at each timepoint was small (median = 2) (Supplementary Figure S4), the expected effect of this factor was small. A large-scale analysis of sampling of mosquitoes across approximately 20 communities in Mali is currently underway, with the investigation of ELISA-based techniques better suited to lower-resource laboratories to further elucidate the temporal and spatial spread of the virus across the country using this approach.

## Conclusions

The congruence of our results based on the serological analysis of blood-fed mosquitoes with conventional serological studies (8, 10) and with active infection records based on PCR carried out in Bamako (Figure 1) lend strong support for the utility of this approach. Akin to wastewater-based epidemiology (41), this non-invasive blood sampling is a promising tool to monitor populations in areas where robust serological data from human subjects is unlikely to be available and where human-biting mosquitoes are common, as is the case in many tropical remote communities. While these population-targeted techniques should be thought of as complementary to and distinct from direct serological studies on human populations, they have been proven to be relevant and useful for public health (community-wide) decision making (41, 42). Understanding exposure rates to pathogens in remote communities as well as changes in reactivity over time are important components of an early warning system targeting remote tropical communities, especially for rare and emerging conditions where conventional surveillance may be considered too costly. Combined with the identification of the blood source, blood-fed mosquito analysis may also be useful to monitor pathogen exposure rates in both human and animal hosts, even if these hosts are poorly characterized (i.e., spillover into an intermediate unknown host). Thus, this technique and other future interrogations of the mosquito bloodmeal could fit well in a one-health paradigm surrounding disease transmission throughout the home or screening across a diverse set of potential reservoirs.

## Data Availability

The raw data supporting the conclusions of this article will be made available by the authors, without undue reservation.

## References

[1] Hasell J Mathieu E Beltekian D Macdonald B Giattino C Ortiz-Ospina E A cross-country database of COVID-19 testing. Sci Data. (2020) 7(1):345. 10.1038/s41597-020-00688-8 33033256 PMC7545176

[2] The Humanitarian Data Exchange. Mali: coronavirus (Covid-19) city level. (2023). Available at: https://data.humdata.org/dataset/malicoronavirus-covid-19-adm2-city-level (Accessed September 14, 2023).

[3] Amanat F Stadlbauer D Strohmeier S Nguyen THO Chromikova V McMahon M A serological assay to detect SARS-CoV-2 seroconversion in humans. Nat Med. (2020) 26(7):1033–6. 10.1038/s41591-020-0913-5 32398876 PMC8183627

[4] Klumpp-Thomas C Kalish H Drew M Hunsberger S Snead K Fay MP Standardization of ELISA protocols for serosurveys of the SARS-CoV-2 pandemic using clinical and at-home blood sampling. Nat Commun. (2021) 12(1):113. 10.1038/s41467-020-20383-x 33397956 PMC7782755

[5] Stadlbauer D Amanat F Chromikova V Jiang K Strohmeier S Arunkumar GA SARS-CoV-2 seroconversion in humans: a detailed protocol for a serological assay, antigen production, and test setup. Curr Protoc Microbiol. (2020) 57(1):e100. 10.1002/cpmc.100 32302069 PMC7235504

[6] Emmerich P Murawski C Ehmen C von Possel R Pekarek N Oestereich L Limited specificity of commercially available SARS-CoV-2 IgG ELISAs in serum samples of African origin. Trop Med Int Health. (2021) 26(6):621–31. 10.1111/tmi.13569 33666297 PMC8014856

[7] Ndaye AN Hoxha A Madinga J Mariën J Peeters M Leendertz FH Challenges in interpreting SARS-CoV-2 serological results in African countries. Lancet Glob Health. (2021) 9(5):e588–9. 10.1016/S2214-109X(21)00060-7 33609481 PMC7906714

[8] Woodford J Sagara I Dicko A Zeguime A Doucoure M Kwan J Severe acute respiratory syndrome coronavirus 2 seroassay performance and optimization in a population with high background reactivity in Mali. J Infect Dis. (2021) 224(12):2001–9. 10.1093/infdis/jiab498 34612499 PMC8522418

[9] Woodford J Sagara I Kwan J Zaidi I Dicko A Duffy PE. Assessing and minimizing the effect of malaria on SARS-CoV-2 serodiagnostics. Front Trop Dis. (2021) 2:781586. 10.3389/fitd.2021.781586

[10] Sagara I Woodford J Kone M Assadou MH Katile A Attaher O Rapidly increasing severe acute respiratory syndrome coronavirus 2 seroprevalence and limited clinical disease in 3 Malian communities: a prospective cohort study. Clin Infect Dis. (2021) 74(6):1030–8. 10.1093/cid/ciab589 PMC8394825 34185847

[11] Tandina F Doumbo O Yaro AS Traore SF Parola P Robert V. Mosquitoes (Diptera: Culicidae) and mosquito-borne diseases in Mali, West Africa. Parasit Vectors. (2018) 11(1):467. 10.1186/s13071-018-3045-8 30103823 PMC6090629

[12] Hall MH Dutro SM Klowden MJ. Determination by near-infrared reflectance spectroscopy of mosquito (Diptera: Culicidae) bloodmeal size. J Med Entomol. (1990) 27(1):76–9. 10.1093/jmedent/27.1.76 2299659

[13] Grubaugh ND Sharma S Krajacich BJ Fakoli LS III Bolay FK Diclaro JW II Xenosurveillance: a novel mosquito-based approach for examining the human-pathogen landscape. PLoS Negl Trop Dis. (2015) 9(3):e0003628. 10.1371/journal.pntd.0003628 25775236 PMC4361501

[14] Barbazan P Palabodeewat S Nitatpattana N Gonzalez JP. Detection of host virus-reactive antibodies in blood meals of naturally engorged mosquitoes. Vector Borne Zoonotic Dis. (2009) 9(1):103–8. 10.1089/vbz.2007.0242 18973442

[15] Contreras CE Beier JC. Detection of human antibodies against plasmodium falciparum antigens in blood meals of anopheline mosquitoes. J Am Mosq Control Assoc. (1992) 8(3):252–5. . 1402862

[16] Cunningham MP Harley JM Southon HA Lumsden WH. Detection of antibodies in blood meals of hematophagous Diptera. Science. (1962) 138(3536):32–3. 10.1126/science.138.3536.32 13882650

[17] Classen DC Morningstar JM Shanley JD. Detection of antibody to murine cytomegalovirus by enzyme-linked immunosorbent and indirect immunofluorescence assays. J Clin Microbiol. (1987) 25(4):600–4. 10.1128/jcm.25.4.600-604.1987 3033015 PMC266042

[18] Lardeux F Torrico G Aliaga C. Calculation of the ELISA's cut-off based on the change-point analysis method for detection of *Trypanosoma cruzi* infection in Bolivian dogs in the absence of controls. Mem Inst Oswaldo Cruz. (2016) 111(8):501–4. 10.1590/0074-02760160119 27384081 PMC4981115

[19] Krajacich BJ Sullivan M Faiman R Veru L Graber L Lehmann T. Induction of long-lived potential aestivation states in laboratory An. Gambiae mosquitoes. Parasit Vectors. (2020) 13(1):412. 10.1186/s13071-020-04276-y 32787948 PMC7424682

[20] Frick H Chow F Kuhn M Mahoney M Silge J Wickham H. rsample: general resampling infrastructure (2022). Available at: https://rsample.tidymodels.org. (Accessed September 14, 2023).

[21] Kuhn M Vaughan D Hvitfeldt E. yardstick: tidy characterizations of model performance (2022). Available at: https://yardstick.tidymodels.org/. (Accessed September 14, 2023).

[22] Ouso DO Otiende MY Jeneby MM Oundo JW Bargul JL Miller SE Three-gene PCR and high-resolution melting analysis for differentiating vertebrate species mitochondrial DNA for biodiversity research and complementing forensic surveillance. Sci Rep. (2020) 10(1):4741. 10.1038/s41598-020-61600-3 32179808 PMC7075967

[23] SAS. SAS/STAT® 9.4: user's guide. Cary, NC: SAS Institute, Inc (2019).

[24] Cade BS Noon BR. A gentle introduction to quantile regression for ecologists. Front Ecol Environ. (2003) 1(8):412–20. 10.1890/1540-9295(2003)001[0412:AGITQR]2.0.CO;2

[25] Couch SP Bray AP Ismay C Chasnovski E Baumer BS Çetinkaya-Rundel M. infer: an {R} package for tidyverse-friendly statistical inference (2021). Available at: https://scholarworks.smith.edu/sds_facpubs/45?utm_source=scholarworks.smith.edu%2Fsds_facpubs%2F45&utm_medium=PDF&utm_campaign=PDFCoverPages. (Accessed September 14, 2023).

[26] Sempos CT Tian L. Adjusting coronavirus prevalence estimates for laboratory test kit error. Am J Epidemiol. (2021) 190(1):109–15. 10.1093/aje/kwaa174 32803245 PMC7454308

[27] Gyawali N Murphy AK Hugo LE Devine GJ. A micro-PRNT for the detection of ross river virus antibodies in mosquito blood meals: a useful tool for inferring transmission pathways. PLoS One. (2020) 15(7):e0229314. 10.1371/journal.pone.0229314 32706777 PMC7380888

[28] Komar N Panella NA Young GR Basile AJ. Methods for detection of West Nile virus antibodies in mosquito blood meals. J Am Mosq Control Assoc. (2015) 31(1):1–6. 10.2987/14-6468R.1 25843170 PMC4785996

[29] Krajacich BJ Huestis DL Dao A Yaro AS Diallo M Krishna A Investigation of the seasonal microbiome of *Anopheles coluzzii* mosquitoes in Mali. PLoS One. (2018) 13(3):e0194899. 10.1371/journal.pone.0194899 29596468 PMC5875798

[30] Antonio-Nkondjio C Simard F Awono-Ambene P Ngassam P Toto JC Tchuinkam T Malaria vectors and urbanization in the equatorial forest region of south Cameroon. Trans R Soc Trop Med Hyg. (2005) 99(5):347–54. 10.1016/j.trstmh.2004.07.003 15780341

[31] Fontenille D Lochouarn L Diatta M Sokhna C Dia I Diagne N Four years’ entomological study of the transmission of seasonal malaria in Senegal and the bionomics of *Anopheles gambiae* and *A. arabiensis*. Trans R Soc Trop Med Hyg. (1997) 91(6):647–52. 10.1016/s0035-9203(97)90506-x 9509170

[32] Beier JC Perkins PV Wirtz RA Koros J Diggs D Gargan TP 2nd Bloodmeal identification by direct enzyme-linked immunosorbent assay (ELISA), tested on anopheles (Diptera: Culicidae) in Kenya. J Med Entomol. (1988) 25(1):9–16. 10.1093/jmedent/25.1.9 3357176

[33] Dambach P Schleicher M Korir P Ouedraogo S Dambach J Sié A Nightly biting cycles of anopheles species in Rural Northwestern Burkina Faso. J Med Entomol. (2018) 55(4):1027–34. 10.1093/jme/tjy043 29635478 PMC6025195

[34] Mburu MM Mzilahowa T Amoah B Hifundo D Phiri KS van den Berg H Biting patterns of malaria vectors of the lower shire valley, southern Malawi. Acta Trop. (2019) 197:105059. 10.1016/j.actatropica.2019.105059 31194960

[35] Mendis C Jacobsen JL Gamage-Mendis A Bule E Dgedge M Thompson R *Anopheles arabiensis* and *An. funestus* are equally important vectors of malaria in Matola coastal suburb of Maputo, southern Mozambique. Med Vet Entomol. (2000) 14(2):171–80. 10.1046/j.1365-2915.2000.00228.x 10872861

[36] Mahanta B Handique R Dutta P Narain K Mahanta J. Temporal variations in biting density and rhythm of *Culex quinquefasciatus* in tea agro-ecosystem of Assam, India. Southeast Asian J Trop Med Public Health. (1999) 30(4):804–9. . 10928380

[37] Pipitgool V Waree P Sithithaworn P Limviroj W. Studies on biting density and biting cycle of *Culex quinquefasciatus*, say in Khon Kaen city, Thailand. Southeast Asian J Trop Med Public Health. (1998) 29(2):333–6. . 9886123

[38] Van Elslande J Oyaert M Ailliet S Van Ranst M Lorent N Vande Weygaerde Y Longitudinal follow-up of IgG anti-nucleocapsid antibodies in SARS-CoV-2 infected patients up to eight months after infection. J Clin Virol. (2021) 136:104765. 10.1016/j.jcv.2021.104765 33636554 PMC7891078

[39] Norris LC Fornadel CM Hung WC Pineda FJ Norris DE. Frequency of multiple blood meals taken in a single gonotrophic cycle by *Anopheles arabiensis* mosquitoes in Macha, Zambia. Am J Trop Med Hyg. (2010) 83(1):33–7. 10.4269/ajtmh.2010.09-0296 20595474 PMC2912572

[40] Tedrow RE Rakotomanga T Nepomichene T Howes RE Ratovonjato J Ratsimbasoa AC Anopheles mosquito surveillance in Madagascar reveals multiple blood feeding behavior and plasmodium infection. PLoS Negl Trop Dis. (2019) 13(7):e0007176. 10.1371/journal.pntd.0007176 31276491 PMC6663035

[41] Ahmed W Angel N Edson J Bibby K Bivins A O'Brien JW First confirmed detection of SARS-CoV-2 in untreated wastewater in Australia: a proof of concept for the wastewater surveillance of COVID-19 in the community. Sci Total Environ. (2020) 728:138764. 10.1016/j.scitotenv.2020.138764 32387778 PMC7165106

[42] Prado T Fumian TM Mannarino CF Resende PC Motta FC Eppinghaus ALF Wastewater-based epidemiology as a useful tool to track SARS-CoV-2 and support public health policies at municipal level in Brazil. Water Res. (2021) 191:116810. 10.1016/j.watres.2021.116810 33434709 PMC7832254

