# Supplementary material for: Tracking SARS-CoV-2 seropositivity in rural communities using blood-fed mosquitoes: a proof-of-concept study
Source: Front Epidemiol. 2023 Dec 13;3:1243691. doi: 10.3389/fepid.2023.1243691 (PMC10911011; doi:10.3389/fepid.2023.1243691)

**Supplementary Materials**

**Supplemental Figure 1:** Numbers of mosquitoes positive for 1-4 anti-SARS-CoV-2 antigens per volunteer at 0, 5, 10, and 30 hours post-feed. Only VAT had known covid status with a SARS-CoV-2 vaccination. All other volunteers had unknown SARS-CoV-2 infection history. Per antigen positivity is defined by pre-pandemic collected wild mosquitoes that fed naturally on Malian individuals. Individuals VAT, VBT, VDT, VFT, VGT, VIT, VLT, and VMT are considered positive by having multiple 2+ antigen positive mosquitoes across multiple time points. VHT, VCT, VET, and VJT are considered negative due to only 1 or fewer antigen positive mosquitoes at any time point. VKT is considered negative due to 11/12 mosquitoes having <2 antigens positive, but does have one 2-antigen-positive mosquito at the 10 hours time-point.

**
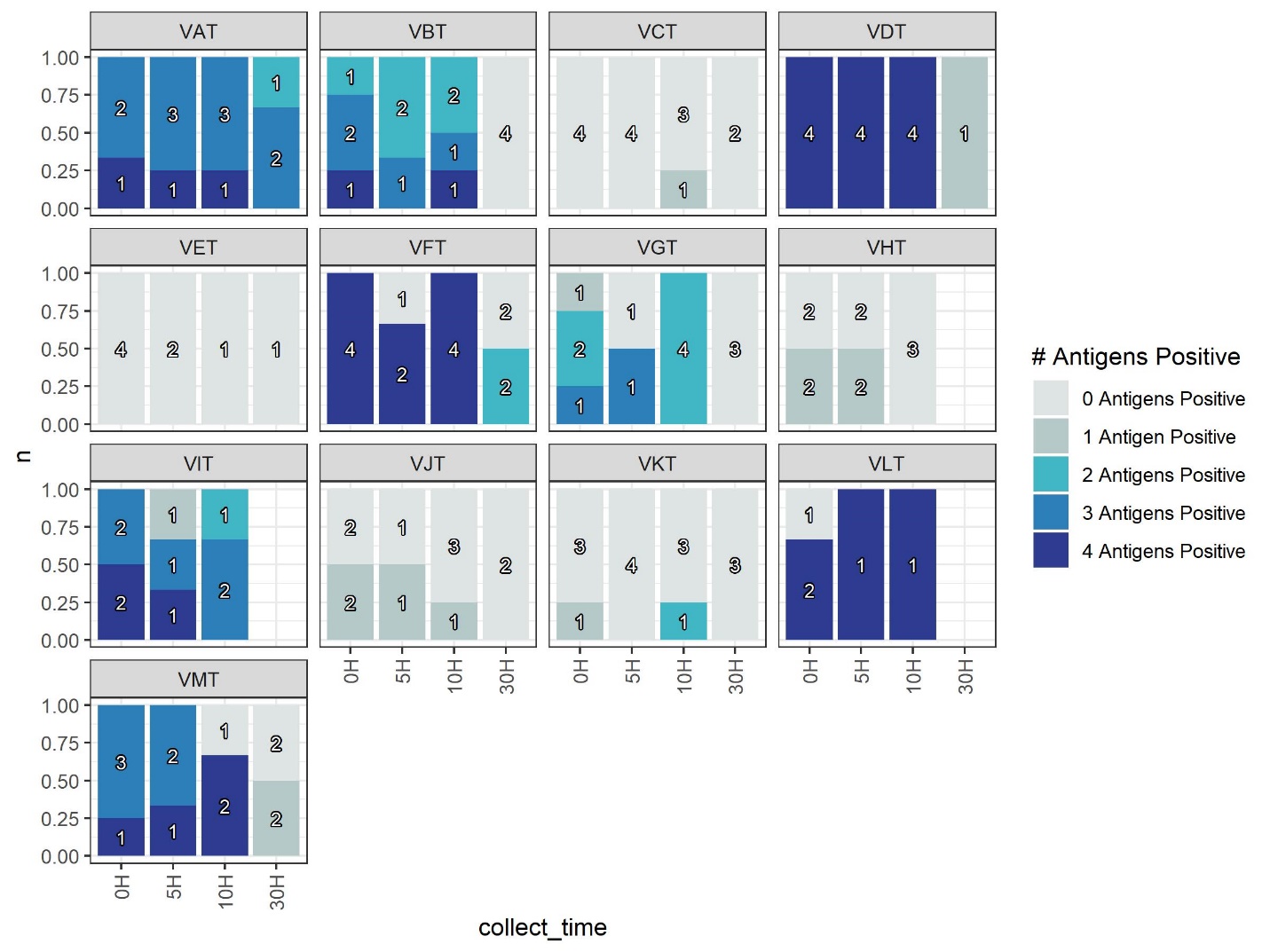
**

**Supplemental Table 1:** Number mosquitoes with the listed number of SARS-CoV-2 antigens above cutoffs per time period.

| **Time Period** | **# Antigens Positive** | **Total tested** |
| --- | --- | --- |
| Pre-pandemic | 0 | 88 |
| Pre-pandemic | 1 | 2 |

**Supplemental figure 2:** Per antigen log2(MFI) values for each volunteer with pre-pandemic cutoffs shown as dashed line per antigen. Number of mosquitoes per positive per antigen/volunteer per time point labeled above boxplots.

**
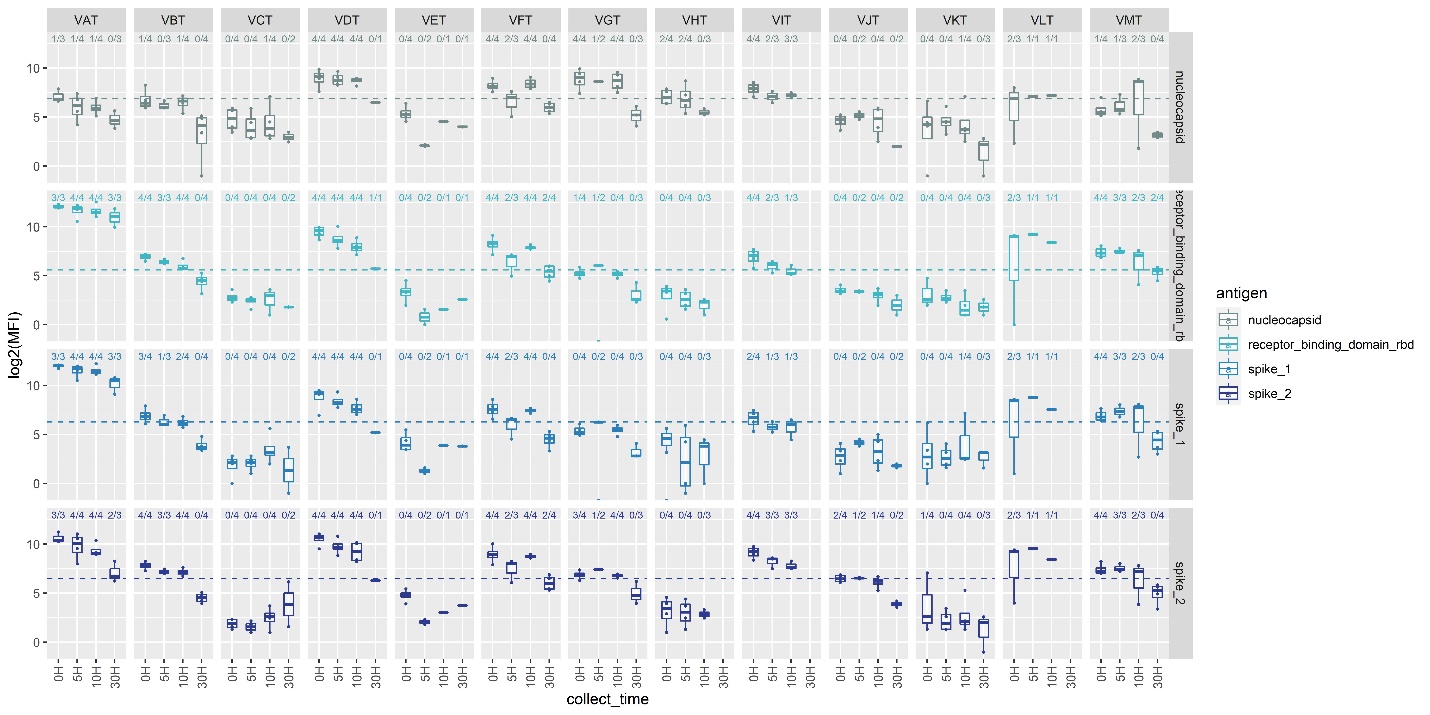
**

**Supplemental figure 3:** Changes in seropositivity over time measured by the number of antigens to which reactivity exceeded cutoff by village
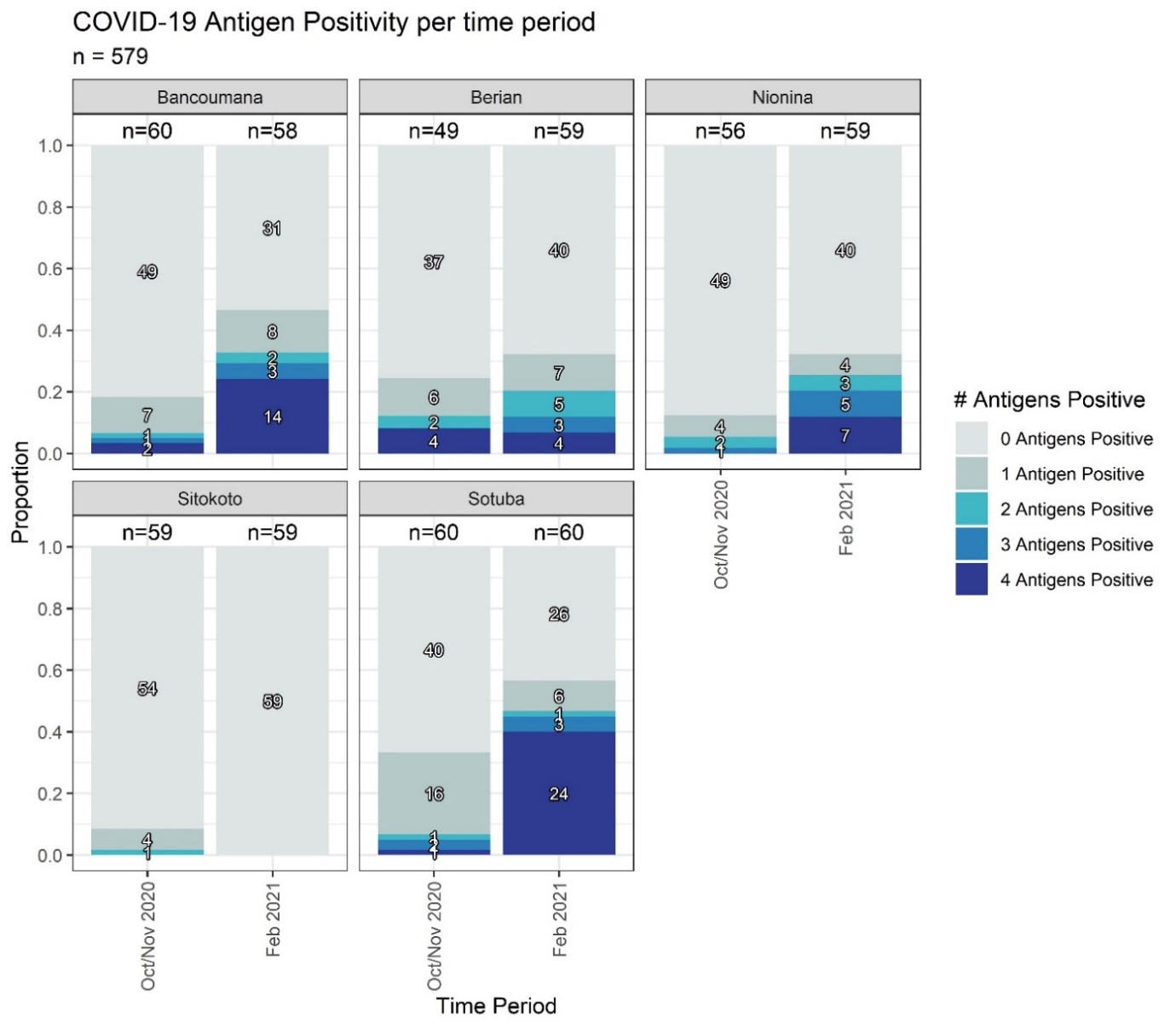


**Supplemental Figure 4:** Relationship between seroprevalence at the mosquito and the house levels (across villages and time points).


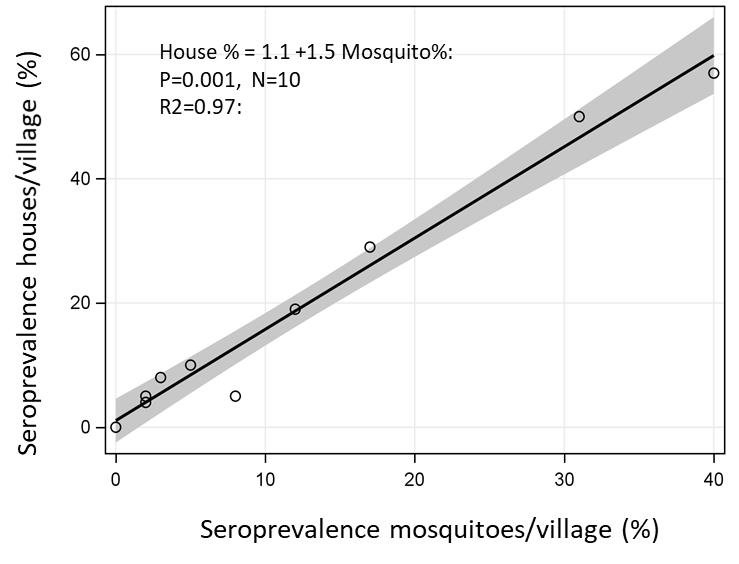


**Supplemental Figure 5:** Blood meal composition across villages and time periods (N=221).


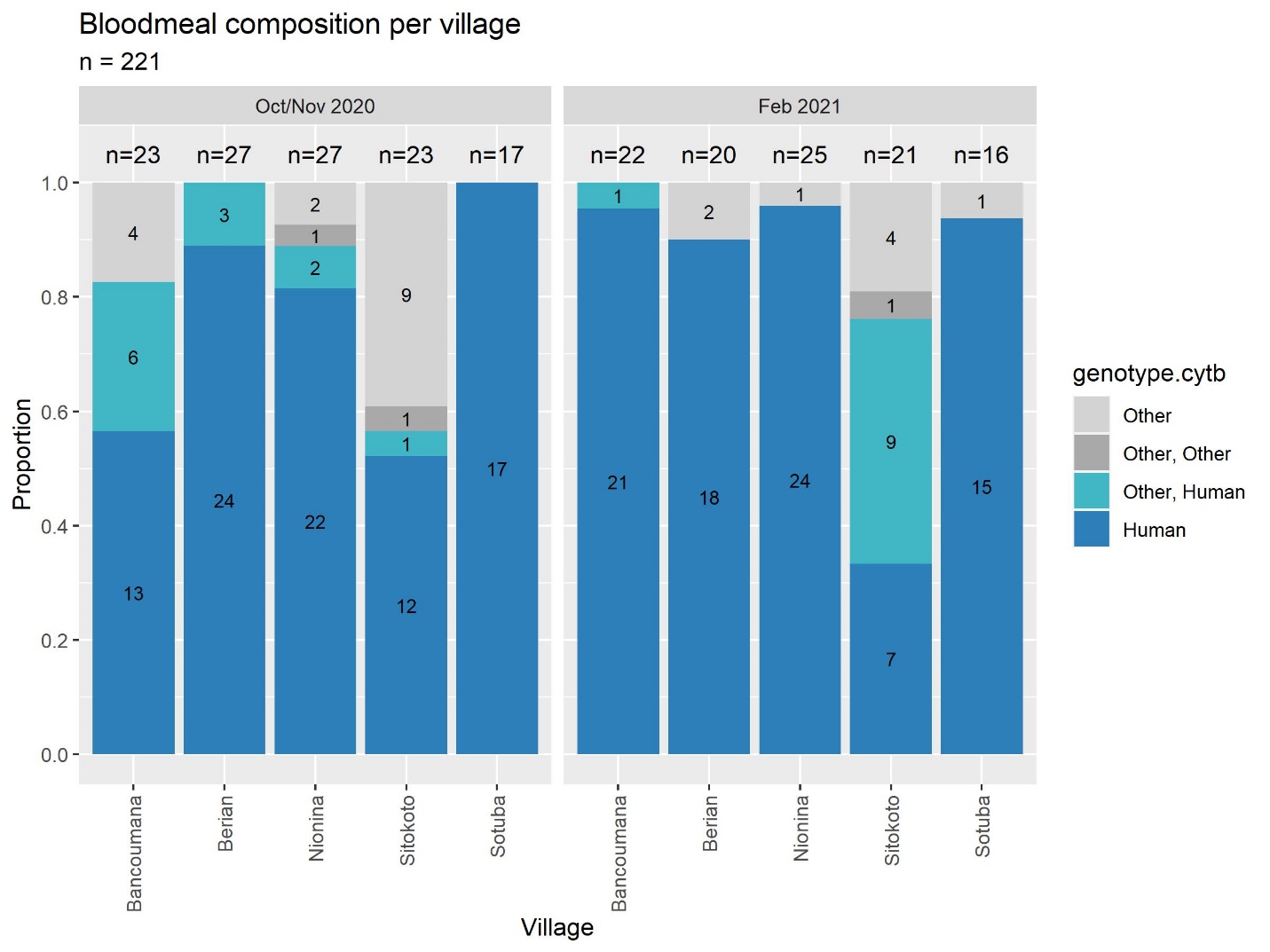


**Supplemental figure 6:** Distribution of the number of mosquitoes collected (green) and analyzed (gold) per house across the five villages by time period (occasionally multiple collection days per time period). Note: the X-axis is not continuous.


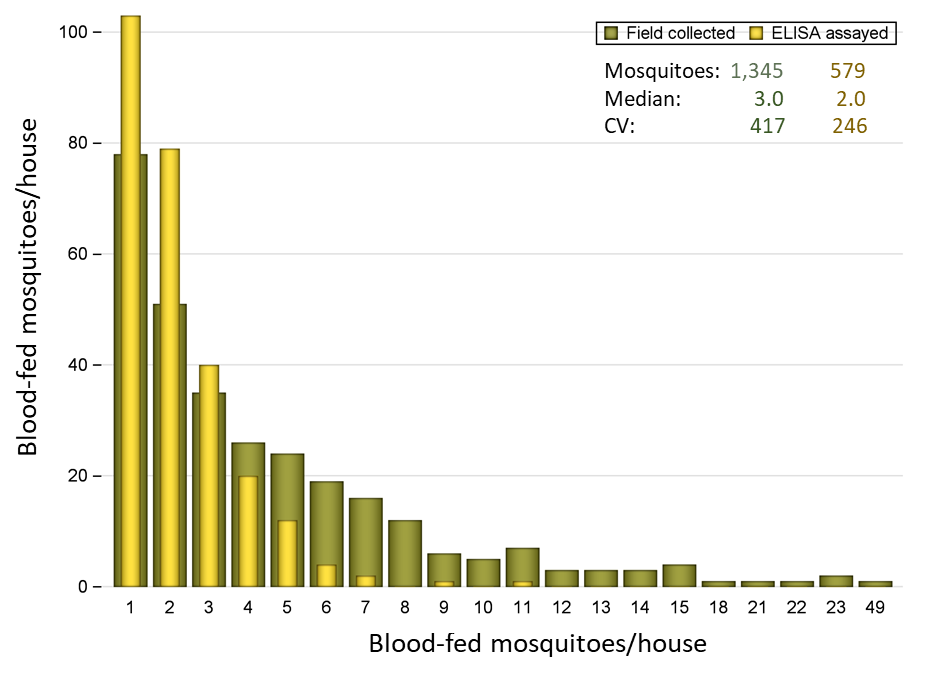

Supplement: Supplementary file 1 [file Datasheet1.docx]
